# Implementing one health in Palestine: Mapping ministerial mechanisms for pandemic preparedness, zoonotic disease control, and inter-sectoral collaboration

**DOI:** 10.1016/j.onehlt.2025.101100

**Published:** 2025-06-05

**Authors:** Leen Humos, Hanin Basha, Walaa Saleh, Fa’ida Awashreh, Ali Baradiea, Eias Salhab, Mohammad Salaymeh, Jakob Zinsstag, Maysaa Nemer, Niveen Abu Rmeileh, Said Abukhattab

**Affiliations:** a Institute of Community and Public Health, Birzeit University, West Bank P.O. Box 14, Palestine; b Swiss Tropical and Public Health Institute, Kreuzstr. 2, CH-4123 Allschwil, Switzerland; c University of Basel, Petersplatz 1, CH-4001 Basel, Switzerland; d Médecins sans frontières OCP, Al Farabi Street 4, Shufat, East Jerusalem, Occupied Palestinian territories; e Department of Public Health, Collage of Health Sciences, Qatar University, Doha, Qatar

**Keywords:** One health, Pandemic preparedness, Zoonotic diseases, Palestine, Inter-sectoral collaboration, Surveillance system

## Abstract

Each year, zoonotic infections result in millions of deaths globally. In Palestine, economic constraints and political instability are challenges that obstruct the management of zoonotic diseases. The main goal of the article is to enhance the preparedness and response capabilities for future health emergencies and pandemics, with zoonotic diseases as a prime example, in Palestine, by adopting the One Health approach. A mixed-method study design was used to meet the study objectives. The quantitative aspect, included two phases: first, an expert survey was conducted to prioritize a list of the most important zoonotic pathogens of national importance in Palestine, followed by a multi-stakeholders group discussion that determined the highest priority zoonotic diseases using a 5-criteria quantitative tool. For the qualitative aspect, we conducted a transdisciplinary, multi-stakeholder group discussion to map the ministerial mechanisms for managing zoonotic diseases. As main findings we identified 43 reported zoonotic diseases, 23 of which were high priority. In addition, this research highlighted key gaps in the existing infrastructure, which are lack of regular screening, weak passive surveillance, irregular health education, weaknesses in communication and follow-up, and limited preparedness for epidemics and pandemics. The One Health approach offers a potential incremental benefit in terms of reducing time to detection, reduction of exposure and cumulative societal cost of outbreaks. It is a promising strategy to bridge the gap between various sectors and lay the groundwork for sustainable and effective management of zoonotic diseases.

## Graphical abstract

**Figure:**
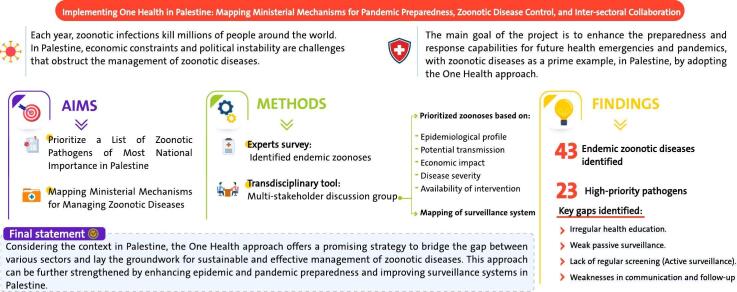

## 1 Introduction

Zoonotic Diseases (ZDs) pose a major threat to global health security [1,2]. ZDs can be transmitted from animals to humans or vice versa and also through vectors [1,2]. It is estimated that 60 % of human infectious diseases are zoonotic in origin [1,3] and 60–75 % of newly emerging diseases are multi-host zoonoses [[3], [4], [5], [6], [7]]. ZDs lead to 2.5 billion cases of human morbidity [2,5] and 2.7 billion cases of mortality annually [2]. Over the last two decades, the globe has faced numerous ZDs outbreaks, including some with recurring outbreaks and others that have led to pandemics [2]. While all regions face the threat of zoonotic diseases, the Eastern Mediterranean Region (EMR) is particularly vulnerable due to limited resources, political unrest, human migration, weak surveillance systems, and limited cross-sector collaboration [6]. The most prevalent zoonotic diseases in the Middle East include endemic illnesses like brucellosis, anthrax, and rabies, as well as epidemic outbreaks of diseases such as Crimean-Congo hemorrhagic fever and Rift Valley fever, and emerging threats such as MERS-CoV and avian influenza [8,9].

To overcome these burdens and to optimize the health of humans and animals, a One Health framework is required [1,6,8]. This integrated, unifying approach aims to sustainably balance and optimize the health of people, animals and ecosystems. Recognizing the health of humans, domestic and wild animals, plants, and the wider environment (including ecosystems) are closely linked and inter-dependent [10,7]. This approach operates at local, national, and international levels to address interrelated public health issues, such as infectious diseases, endemic zoonotic, neglected tropical and vector-borne diseases, antimicrobial resistance, and food safety [7,11]. One Health is a leading integrated concept of health of all species and is rapidly gaining attention by governments, regional organizations such as the G7 [12], and international organizations [13]. Despite its significance, operationalization of One Health remains slow in many countries due to various factors [3,6], including inadequate understanding of the concept [14], lack of financial resources [11], and the absence of capacity-building programs [6].

The adoption of a One Health approach in Palestine is hindered by inadequate resources and limited policy support for multi-sectoral cooperation [8,14]. The chronic conflict has resulted in overcrowding, unsanitary living conditions, lack of environmental sanitation and outbreaks of infectious diseases [[15], [16], [17], [18]]. This situation is further exacerbated by the broader pre-existing socio-economic and environmental situation in the country [8]. A strong integrated surveillance system (iSRS) [19] is essential for eliminating ZDs' effects on human and animal (5, 12). In Palestine, current surveillance systems are fragmented with inconsistent protocols and insufficient data collection (8, 10, 13).

In this context, One Health approach is critically needed to address ZDs and to improve preparedness for zoonotic outbreaks. This study aims to comprehensively prioritize a list of zoonotic pathogens in Palestine, review ministerial structures for disease control, and emphasize the need for inter-sectoral collaboration to strengthen the overall surveillance system in Palestine.

## 2 Methods

**Fig. 1:**
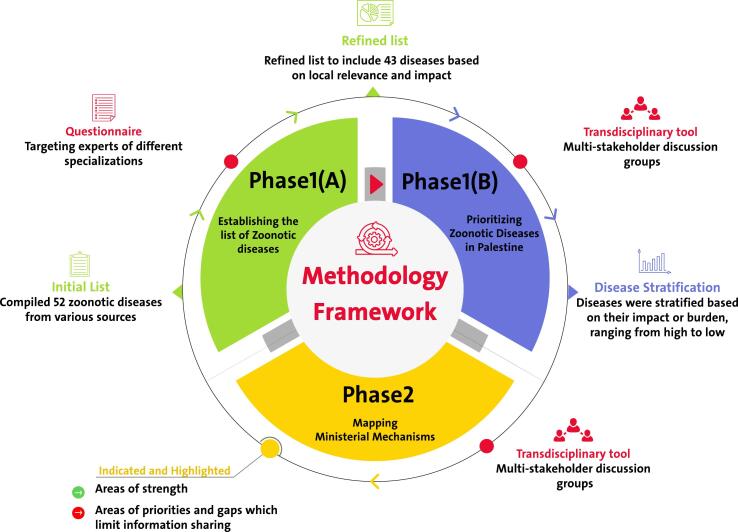
Methodology Framework.

A mixed methods approach was used to capture the attitudes, views, opinions, interests, and needs of all relevant stakeholders. The study facilitated exchanges among participants, promoting coordination and cooperation. This was achieved through a combination of multi-stakeholder discussion groups and a structured questionnaire. An overview of the methodological framework is presented in Fig. 1.

### 2.1 Phase 1: prioritizing zoonotic diseases

A total of 36 Palestinian infectious disease experts, representing various sectors (see Supplementary Material 1), were invited via email to participate in the study. The email included a project overview and a link to an online survey form listing the ZDs, where the experts were asked to identify the endemic zoonotic diseases in Palestine. Each expert evaluated the presence of each zoonotic disease by selecting on of the following: “Yes” (present), “No” (not present), or “not familiar” (with the diseases). The response frequency was calculated for each ZD and ranked descending starting with the disease with the highest “Yes” responses. Based on these expert assessments, conducted between July and August 2023, diseases not considered prevalent in Palestine were removed from the list.

The list of ZDs identified by experts was prioritized using a transdisciplinary approach [20,21]. This process involved multi-stakeholder discussion groups and was conducted through round table discussions during a workshop held in August 2023. Key stakeholders of representatives, technical experts, and decision-makers attended and were grouped based on their profession to obtain consistent responses. Participant details in the meeting is described in Supplementary Material S1. The CDC's One Health Zoonotic Disease Prioritization tool, a semi-quantitative selection tool, was adapted and modified to align with the specific context of Palestine for the prioritization phase [22]. This tool ranks diseases based on five criteria: epidemiological profile, disease severity, availability of interventions, transmission potential, and socio-economic and environmental impact [22]. Each criterion had a predefined weight, initially determined based on literature standards and subsequently refined through expert consensus, with each response given a corresponding score. The questions used for this process were derived from relevant sources [[23], [24], [25]], and are presented along with the weighted score calculation formula in Supplementary Material 2.

### 2.2 Phase 2: existing structures regarding zoonotic disease reporting and control

To evaluate the reporting, surveillance, response, prevention, and control of Palestine's most prevalent ZDs, a transdisciplinary tool, a multi-stakeholder discussion groups were held in April 2024. A total of 23 experts from Ministries, local World Health Organization (WHO), The United Nations Relief and Works Agency (UNRWA), Food and Agriculture Organization (FAO), and other academic institutions attended these roundtable discussions. Participants were divided into two groups, each structured to include representatives from all key sectors and stakeholder types—medical (physicians and paramedics), veterinary, academic, environmental, governmental, NGO, and private. This setup reduced group size to encourage active participation, allowed for focused, sector-specific discussions, and minimized dominance by any one discipline. It also helped ensure consistent, comparable, and reliable outputs. Two moderators were present in each room to guide the discussion. At the end, the two groups gathered in one room to present and discuss the results of the two frameworks. We were able to map the infrastructure of existing networks of communication, collaboration, and coordination across the sectors. We assessed the surveillance system and case management protocols in the MOH and MOA using scenario-based discussions. Furthermore, we examined the links between public health and veterinary sectors in terms of communication to identify strengths and gaps. Additionally, two scenario-based interviews were conducted in September 2024 with experts from the private sector, a healthcare professional and a veterinarian, focusing on Brucellosis. The interviews explored how the reporting system functions across governmental and private sectors by presenting scenarios related to brucellosis outbreaks. Experts were asked about reporting procedures, data-sharing mechanisms, and the challenges of coordinating responses between governmental and private entities.

## 3 Results and discussion

### 3.1 Zoonotic disease prioritization

#### 3.1.1 Zoonotic diseases profile in Palestine

Based on the knowledge and experience of 25 infectious disease experts (see Supplementary Material 1 for professions), 43 zoonotic diseases were identified in Palestine. The figure in supplementary Material 3 illustrates the list of diseases and the percentage distribution of experts' responses on each disease.

#### 3.1.2 Prioritizing zoonotic diseases

Twenty-three pathogens scored above “0.5” and were identified as high-priority pathogens, with significant concerns in Palestine. The highest-scoring pathogen was identified to be *Brucella*, with a score of “0.793”. Avian influenza and Rabies, ranked second and third, with scores of “0.750” and “0.730” respectively. Followed by *Listeria* “0.641”, *Echinococcus* “0.640”, *Coxiella brunetti* “0.62” and *Leishmania* “0.593”. All of these pathogens pose serious threats to human health, with high case-fatality rates, potential for widespread transmission, and significant economic burden due to high treatment costs or the need for external interventions [4,26]. All pathogens and their scores are available in Table 1.

**Table 1:** Weights for ZDs in Palestine utilizing the prioritization tool.

Disease	Score	Disease	Score
Brucellosis	**0.794**	Leptospirosis	**0.510**
Avian influenza	**0.751**	Plague (*Yersinia pestis*)	**0.486**
Rabies	**0.731**	Cryptococcosis	**0.486**
Listeriosis	**0.641**	Ebola	**0.469**
Echinococcosis	**0.640**	Cryptosporidiosis (*Cryptosporidium parvum)*	**0.463**
Q Fever (*Coxiella brunetti*)	**0.603**	Amebiasis (*Entameba histolytica*)	**0.462**
Leishmaniasis	**0.594**	*Giardia lamblia*	**0.455**
Salmonellosis	**0.578**	Tularemia (*Francisella tularensis*)	**0.450**
Anthrax	**0.568**	Lyme disease (*Borrelia burgdorfei*)	**0.450**
Bovine Tuberculosis (*Mycobacterium bovis)*	**0.564**	Ringworm	**0.426**
Taeniasis	**0.562**	Hookworms	**0.425**
Toxoplasmosis (*Toxoplasma gondii*)	**0.559**	*Streptococcus*	**0.422**
Dengue	**0.555**	Rift valley fever	**0.398**
Rocky Mountain spotted fever (*Rickettsia rickettsii*)	**0.550**	Bartonellosis (*Bartonella*)	**0.390**
West Nile Virus	**0.547**	Yellow fever virus	**0.360**
MRSA (*Staphylococcus aureus*)	**0.542**	Eastern equine encephalitis virus	**0.351**
Cysticercosis	**0.535**	Histoplasmosis	**0.343**
Campylobacteriosis	**0.533**	*Trypanosoma*	**0.341**
Anaplasmosis	**0.531**	*Trichinella*	**0.340**
*Escherichia coli*	**0.530**	Hepatitis E	**0.306**
*Klebsiella pneumoniae*	**0.519**	Western encephalitis virus	**0.260**
MERS-CoV	**0.510**	*Ehrlichia*	**0.240**

Alignment with the Palestinian Annual Health Reports for years 2021 and 2022 was only found for *Brucella* [22,27]. These reports confirm *Brucella* as endemic in Palestine, with confirmed and documented prevalence. A Libyan study similarly highlighted *Brucella* as a significant zoonotic threat, underscoring its endemic status and public health impact in the region [28]. Moreover, our results align with the results of K.A. Kheirallah et al. in Jordan, where Rabies, Avian Influenza, and Brucellosis were ranked among high-priority diseases [29]. *Coxiella burnetti* and *Leishmania* were among the high-priority pathogens, despite having a lower score, in the findings of McAlester et al., aligning with our results [30]. As for Avian Influenza, the highly pathogenic virus is of serious concern in both Egypt and Israel [31].

Furthermore, the direct and indirect health impacts of the diseases, as well as the availability of relevant medical interventions, were discussed during the workshop. One of the participants mentioned that *“although treatments and vaccines are available for some diseases, such as Brucella, many other diseases lack their corresponding treatments”*. Another participant emphasized that “*Even if treatments are available, sometimes they are ineffective or unaffordable”*. These statements highlight the significance of prioritizing ZDs, as it enables us to focus on high priority pathogens, thus, directing our limited capacities and resources to tackle the most critical ones.These statements highlight the need for active surveillance as a strategy for comprehensive disease prevention rather than treatment [32,33].

Moreover, the workshop discussed how collaborations for handling zoonosis vary depending on the pathogen. As the participants agreed, some pathogens are handled by the MoH, while others are handled by the MoA. Collaborations between both MoH and MoA were confirmed for specific pathogens, but not all. Additionally, municipalities were involved in some pathogens. This point highlights the need to enhance cross-sectoral coordination and collaboration for effective management and control of diseases [34,35].

### 3.2 Mapping analysis: gaps, challenges, and limitations

#### 3.2.1 Identifying strengths and gaps in the current structures

**Fig. 2:**
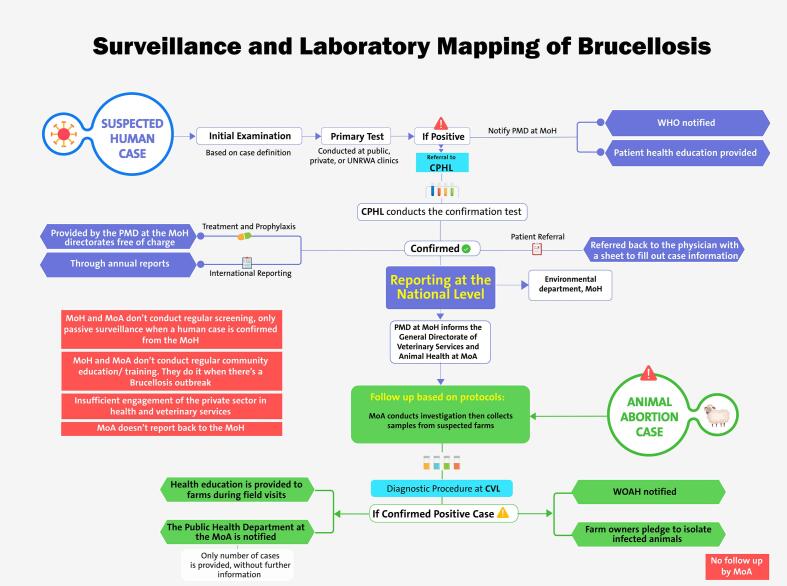
Surveillance and laboratory mapping of brucellosis; MoH: Ministry of Health, CPHL: Central Public Health Laboratory, PMD: Preventive Medicine Department, MoA: Ministry of Agriculture, CVL: Central Veterinary Laboratory, WHO: World Health Organization, and WOAH: World Organization for Animal Health.

**Fig. 3:**
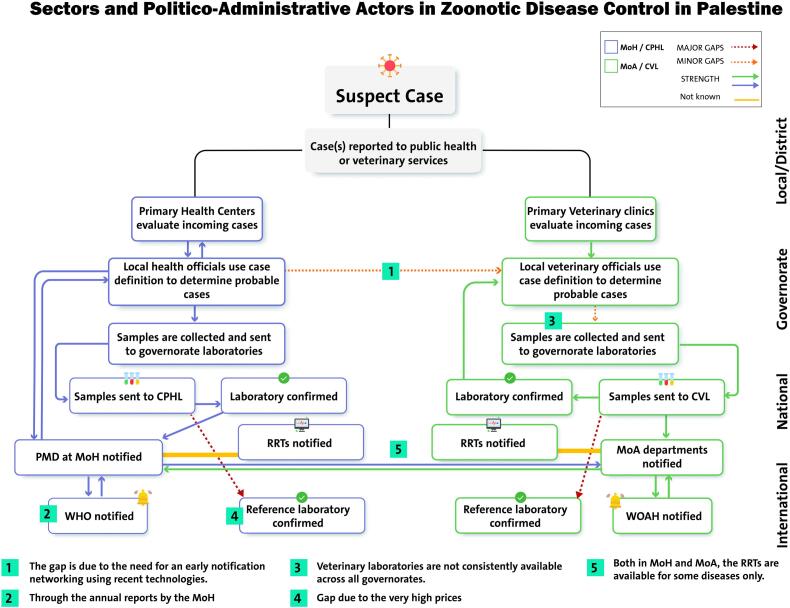
Sectors and politico-administrative actors in zoonotic disease control in Palestine; MoH: Ministry of Health, CPHL: Central Public Health Laboratory, PMD: Preventive Medicine Department, MoA: Ministry of Agriculture, CVL: Central Veterinary Laboratory, RRT: Rapid Response Team, WOAH: World Organization for Animal Health, and WHO: World Health Organization.

Scenario-based discussions among stakeholders provided a comprehensive assessment of the system, identifying both strengths and critical gaps. The system demonstrates several strengths, including a structured process for sample collection and testing through national laboratories, along with integrated case evaluation in both health and veterinary sectors using standardized case definitions. It also supports international coordination by facilitating communication with organizations like WHO and WOAH, enhancing global collaboration in managing and controlling zoonotic diseases. However, discussions among stakeholders highlighted significant gaps in the current surveillance system, inter-sectoral collaboration and communication, the availability of Rapid Response Teams (RRTs), and the lack of adequate human resources and laboratory facilities, as illustrated in Fig. 2, Fig. 3.

Stakeholders raised the issue that the national annual health reports may not fully reflect the reported incidence of all zoonotic disease cases in the country. The underreporting of cases is primarily attributed to the passive nature of the current surveillance system. This highlights the importance of adopting a robust integrated surveillance–response system (iSRS) [36,37] that incorporates human and animal health, the environment, and food production components [38,39].

Regarding communication and coordination, strong intra-sectoral communication exists within each ministry. However, stakeholders addressed delays in inter-sectoral communication and reporting when a probable case is determined. This negatively impacts timely and effective responses to such cases [35,40]. Therefore, to enhance inter-sectoral communication, stakeholders proposed using computerized health information systems for timely notifications rather than delayed paperwork. This has been supported by evidence following the COVID-19 pandemic, which demonstrated the effectiveness of electronic health data in surveillance [41]. Additionally, they recommended that both ministries share updated reports monthly to maintain continuous communication and collaboration.

Despite the clear responsibilities and assigned roles for MoA and MoH, consistent inter-sectoral collaboration is often lacking in the control and management of ZDs. This problem is also apparent in the Jordanian infrastructure [25], suggesting a potential regional challenge in this regard. Moreover, lack of communication and collaboration with the private sector has resulted in minimal involvement and engagement in addressing ZDs and veterinary services, which is similar to the situation in Jordan [25].

Although efforts are made by relevant health and agricultural authorities to control ZDs, consistent and effective inter-sectoral collaboration often remains limited. The lack of permanent Rapid Response Teams (RRTs) in both ministries creates additional burdens and negatively impacts staff's productivity. To address these challenges, stakeholders have emphasized the importance of operationalizing the One Health approach by strengthening communication and collaboration across sectors, investing in human and resource capacity, and establishing dedicated RRT units [42]. Establishing a national One Health committee consisting of representatives from several ministries and other sectors, would assist in overcoming the identified challenges in early detection, rapid response, and long-term monitoring of zoonotic diseases (30).

While health education is provided to farmers during field visits, stakeholders expressed the need to regularly address poor awareness and limited knowledge regarding ZDs among relevant staff, not only in times of confirmed cases. Underscoring the crucial need for staff capacity building for effective management of emerging ZDs. This is achieved by strengthening staff's awareness and education through continuous training programs [39,40].

Furthermore, most governorates in Palestine lack veterinary laboratory facilities, with only a few locations equipped to provide such services. Consequently, veterinary cases are often referred to distant laboratories, which is both inconvenient and impractical. Furthermore, diagnostic laboratories for certain zoonotic diseases are not available in Palestine, and some diseases require a confirmatory process in specialized reference laboratories, including Biosafety Level 3 (BSL-3) laboratories, which are also absent in Palestine. Thus, samples are sent to referral laboratories in the region. This process if often hindered by logistical difficulties, economic constraints, as well as political instability. That's why, despite the high cost in the short term, investing in a well-equipped reference laboratory for all diseases in the country would save money in the long term [39].

### 3.3 Methods to assess zoonotic disease surveillance systems in conflict zones

Palestine's existing health system is greatly affected by political instability, insufficient infrastructure and governance issues marked by fragmented regulations and limited cohesion [43,44]. The Palestinian health system is emergency-oriented, with limited resources, inadequate financing, and lacking inter-sectoral coordination [43,44]. Challenges like fragmented data collection have been highlighted in areas such as adolescent health, maternal and child health, and food safety [39,45,46]. Therefore, pandemic and epidemic preparedness and the availability of accurate, real-time data are hindered in the Palestinian context. This study's methodology addressed these limitations by incorporating specific strategies, such as involving local experts from various sectors throughout the research process. Workshops were conducted with representative from ministries, NGOs, academia and other experts provided a realistic understanding of the infrastructure, allowing for targeted recommendations. Libya employed a similar approach, highlighting gaps in diagnostic capacities and inter-sectoral coordination. This methodology not only attempts to fill existing gaps and improve preparedness but also offers a reproducible framework for similar regions facing comparable resource constraints [28].

### 3.4 One health in Palestine

The critical need for One Health to become the comprehensive approach for combatting ZDs globally is amplified in countries with prolonged and intense conflicts, like Palestine. The ongoing political instability in Palestine exacerbates health challenges by fragmenting the health sector and negatively impacting social and community structures and, consequently, economic progress. This, coupled with the limited resources, underscores the need for One Health in the country. Adopting this approach in Palestine would enhance and strengthen the available passive surveillance system, promote economic growth, and assist in predicting and preventing public health threats [39,44,47,48].

Palestine can draw on existing One Health evidence for the control and elimination of zoonotic diseases. For example the mass vaccination livestock against brucellosis is highly profitable from a societal perspective with a benefit-cost ratio of 3 to 1 [49]. Similarly the coordinated mass vaccination of dogs leads to the elimination of rabies with high human welfare benefits and an overall benefit-cost ratio exceeding 10 to 1 [50]. National control and elimination programs can build up on this evidence but will require coordination with neighboring countries.

## 4 Conclusion

This study represents an initial step towards prioritizing ZDs in Palestine and mapping ministerial mechanisms for reporting and controlling ZDs. The research engaged all levels of health and agriculture-related agencies across the West Bank involved in controlling the most vital ZDs. Initially, the study identified crucial ZDs in Palestine, collaborating with leading infectious disease specialists. Subsequently, it examined the current system within the MoH and MoA for responding to brucellosis cases and how these two ministries interact and collaborate.

While some pathogens, such *as Brucella* and *Leishmania*, are categorized as endemic in Palestine, the lack of a One Health surveillance system hinders accurate categorization of other pathogens as endemic or sporadic. Therefore, this study serves as an initiative to enhance active integrated surveillance response system in Palestine to determine the status of the uncategorized diseases as endemic or sporadic, which will positively reflect on other public health aspects.

Addressing the existing gaps and developing the current surveillance systems are essential for embracing One Health. Considering the context in Palestine, One Health approach offers a promising strategy by bridging divisions among various sectors, enhancing collaboration, and reducing fragmentation. Furthermore, One Health would lay the ground for sustainable and effective management of zoonotic diseases, as well as enhancing the country's epidemic and pandemic preparedness.

## CRediT authorship contribution statement

**Leen Humos:** Writing – review & editing, Writing – original draft, Visualization, Validation, Resources. **Hanin Basha:** Writing – review & editing, Writing – original draft, Visualization, Formal analysis, Data curation, Conceptualization. **Walaa Saleh:** Writing – original draft, Investigation, Formal analysis, Data curation. **Fa’’ida Awashreh:** Writing – original draft, Investigation, Formal analysis, Data curation. **Ali Baradiea:** Writing – original draft, Investigation, Data curation. **Eias Salhab:** Writing – original draft, Methodology, Data curation. **Mohammad Salaymeh:** Writing – original draft, Data curation. **Jakob Zinsstag:** Writing – review & editing, Validation, Methodology, Funding acquisition, Conceptualization. **Maysaa Nemer:** Writing – review & editing, Formal analysis, Conceptualization. **Niveen Abu Rmeileh:** Writing – review & editing, Methodology, Funding acquisition, Formal analysis, Conceptualization. **Said AbuKhattab:** Writing – review & editing, Writing – original draft, Visualization, Validation, Supervision, Resources, Project administration, Methodology, Investigation, Funding acquisition, Formal analysis, Data curation, Conceptualization.

## Ethical approval

The study proposal and tools were reviewed and approved by the Ethics Review Committee at the Institute of Community and Public Health, Birzeit University (reference number: 2023 (5–2). All data was anonymized and processed in accordance with relevant laws and regulations of the jurisdiction.

## Funding

This work was funded by the Leading House for the Middle East and North Africa (LHMENA), Switzerland (Grant ID: LHMENA RPG-2022-22).

## Declaration of competing interest

None.

## Data Availability

Data will be made available on request.

## Appendix A Supplementary data

Supplementary material1 Image 1

Supplementary material2 Image 2

Supplementary material3 Image 3

Supplementary material4 Image 4

## References

[1] Shaheen M.N.F. (2022). The concept of one health applied to the problem of zoonotic diseases. Rev. Med. Virol..

[2] Erkyihun G., Alemayehu M.B. (2022). One health approach for the control of zoonotic diseases. Zoonoses.

[3] Rahman M.T., Sobur M.A., Islam M.S., Ievy S., Hossain M.J., El Zowalaty M.E. (2020). Zoonotic diseases: etiology, impact, and control. Microorganisms.

[4] Di Bari C., Venkateswaran N., Fastl C., Gabriël S., Grace D., Havelaar A. (2023). The global burden of neglected zoonotic diseases: current state of evidence. One Health.

[5] Carpenter A., Waltenburg M.A., Hall A. (2022).

[6] Mahrous H., Redi N., Nguyen T.M.N., Al Awaidy S., Mostafavi E., Samhouri D. (2020). One Health operational framework for action for the Eastern Mediterranean Region, focusing on zoonotic diseases. East Mediterr. Health J..

[7] Mediterranean World Health Organization (WHO) Regional Office for the Eastern (2022).

[8] Team WHO (2020).

[9] Organization World Health (2025).

[10] (OHHLEP) One Health High-Level Expert Panel, Adisasmito W.B., Almuhairi S., Behravesh C.B., Bilivogui P., Bukachi S.A. (2022). One health: a new definition for a sustainable and healthy future. PLoS Pathog..

[11] (2023). Toward public health resilience in the eastern Mediterranean region: findings from the seventh eastern Mediterranean public health network regional conference. Interact. J. Med. Res..

[12] G7 Carbis Bay Health Declaration Name of Web Site. https://www.gov.uk/government/publications/g7-carbis-bay-health-declaration/g7-carbis-bay-health-declaration.

[13] Zinsstag J., Kaiser-Grolimund A., Heitz-Tokpa K., Sreedharan R., Lubroth J., Caya F. (2023). Advancing one human-environmental-animal health for global health security: what does the evidence say?. Lancet.

[14] Al-Eitan L., Sendyani S., Alnemri M. (2023). Applications of the one health concept: current status in the Middle East. J. Biosaf. Biosecur..

[15] Hussein S., Ahmed S., Qurbani K., Saber A., Essa R. (2024). Infectious diseases threat amidst the war in Gaza. J. Med. Surg. Public Health.

[16] Husseini A. (2024).

[17] Shorrab A., Nassef M., Subhi A., Giwa B., Buheji M. (2024). Health in the crossfire -analyzing and mitigating the multifaceted health risks of the 2023 war on Gaza. Public Health Res..

[18] Kearney J.E., Thiel N. (2024). Conflicts in Gaza and around the world create a perfect storm for infectious disease outbreaks.

[19] Zinsstag J., Utzinger J., Probst-Hensch N., Shan L., Zhou X.N. (2020). Towards integrated surveillance-response systems for the prevention of future pandemics. Infect. Dis. Poverty.

[20] Zinsstag J., Pelikan K., Berger Gonzalez M., Kaiser-Grolimund A., Crump L., Mauti S., Lawrence R.J. (2023). Handbook of Transdisciplinarity: Global Perspective.

[21] Anonymous (2020).

[22] Rist C.L., Arriola C.S., Rubin C. (2014). Prioritizing zoonoses: a proposed one health tool for collaborative decision-making. PLoS One.

[23] Pieracci E.G., Hall A.J., Gharpure R., Haile A., Walelign E., Deressa A. (2016). Prioritizing zoonotic diseases in Ethiopia using a one health approach. One Health.

[24] Wang X., Rainey J.J., Goryoka G.W., Liang Z., Wu S., Wen L. (2019). Using a one health approach to prioritize zoonotic diseases in China. PLoS One.

[25] Abutarbush S., Hamdallah A., Hawawsheh M., Alsawalha L., Elizz N., Dodeen R. (2022). Implementation of one health approach in Jordan: review and mapping of ministerial mechanisms of zoonotic disease reporting and control, and inter-sectoral collaboration. One Health.

[26] Noguera Z., Liz P., Charypkhan D., Hartnack S., Torgerson R.P., Rüegg S.R. (2022). The dual burden of animal and human zoonoses: a systematic review. PLoS Negl. Trop. Dis..

[27] Mackenzie J.S., Jeggo M. (2019). The one health approach-why is it so important?. Trop. Med. Infect. Dis..

[28] Miller L., Elmselati H., Fogarty A., Farhat M., Standley C.J., Abuabaid H.M. (2023). Using one health assessments to leverage endemic disease frameworks for emerging zoonotic disease threats in Libya. PLOS Glob. Public Health.

[29] Kheirallah K.A., Al-Mistarehi A.H., Alsawalha L., Hijazeen Z., Mahrous H., Sheikali S. (2021). Prioritizing zoonotic diseases utilizing the one health approach: Jordan’s experience. One Health.

[30] McAlester J., Kanazawa Y. (2022). Situating zoonotic diseases in peacebuilding and development theories: prioritizing zoonoses in Jordan. PLoS One.

[31] Symochko L., Pereira P., Demyanyuk O., Pinheiro C., M N, D Barcelo. (2024). Resistome in a changing environment: hotspots and vectors of spreading with a focus on the Russian-Ukrainian war. Heliyon.

[32] Sharan M., Vijay D., Yadav J., Bedi J., Dhaka P. (2023). Surveillance and response strategies for zoonotic diseases: a comprehensive review. Sci. One Health.

[33] Kheirallah K., AlMistarehi A., Alsawalha L., Hijazeen Z., Mahrous H., Sheikali S. (2021). Prioritizing zoonotic diseases utilizing the one health approach: Jordan’s experience. One Health.

[34] Kelly T.R., Machalaba C., Karesh W.B., Crook P.Z., Gilardi K., Nziza J. (2020). Implementing one health approaches to confront emerging and re-emerging zoonotic disease threats: lessons from PREDICT. One Health Outlook.

[35] Alimi Y., Wabacha J. (2023). Strengthening coordination and collaboration of one health approach for zoonotic diseases in Africa. One Health Outlook.

[36] Osman Y., Ali S.M., Schelling E., Tschopp R., Hattendorf J., Muhumed A., Zinsstag J. (2021). Integrated community based human and animal syndromic surveillance in Adadle district of the Somali region of Ethiopia. One Health.

[37] Osman Y., Zinsstag J., Abtidon R., Hattendorf J., Crump L., Wali H. (2023). Operationalizing a community-based one health surveillance and response in Adadle district of Ethiopia. CABI One Health.

[38] Abukhattab S., Taweel H., Awad A., Crump L., Vonaesch P., Zinsstag J. (2022). Systematic review and Meta-analysis of integrated studies on Salmonella and Campylobacter prevalence, Serovar, and phenotyping and genetic of antimicrobial resistance in the Middle East—A one health perspective. Antibiotics.

[39] Abukhattab S., Kull M., Abu-Rmeileh N., Cissé G., Crump L., Hattendorf J., Zinsstag J. (2022). Towards a one health food safety strategy for Palestine: a mixed-method study. Antibiotics.

[40] Schwind J., Goldstein T., Thomas K., Mazet J., Smith W. (2014). Consortium Predict.Capacity building efforts and perceptions for wildlife surveillance to detect zoonotic pathogens: comparing stakeholder perspectives. BMC Public Health.

[41] Khubone T., Tlou B., Mashamba-Thompson T.P. (2020). Electronic health information systems to improve disease diagnosis and management at point-of-care in low and middle income countries: a narrative review. Diagnostics (Basel).

[42] Elnosserry S., Buliva E., Abdalla Elkholy A., Mahboob A., Fazaludeen Koya S., Abubakar A. (2024). Rapid response teams in the eastern Mediterranean region: results from the baseline survey of country-level capacities, operations and outbreak response capabilities. Glob. Public Health.

[43] Abuzerr S., Zinszer K., Assan A. (2021). Implementation challenges of an integrated One Health surveillance system in humanitarian settings: a qualitative study in Palestine. SAGE Open Med..

[44] Hamdan M., Defever M., Abdeen Z. (2003). Organizing health care within political turmoil: the Palestinian case. Int. J. Health Plann. Manag..

[45] Shalash A., AbuRmeileh N., Kelly D., Elmusharaf K. (2024). Opportunities and challenges of using a health information system in adolescent health management: a qualitative study of healthcare providers’ perspectives in the West Bank, occupied Palestinian territory. PLoS One.

[46] Alyahya M., Abu-Rmeileh N., Khader Y., Nemer M., Al-Sheyab N., Corbion A. (2022). Maturity level of digital reproductive, maternal, newborn, and child health initiatives in Jordan and Palestine. Methods Inf. Med..

[47] Abukhattab S., Hosch S., Abu-Rmeileh N., Hasan S., Vonaesch P., Crump L. (2023). Whole-genome sequencing for one health surveillance of antimicrobial resistance in conflict zones: a case study of Salmonella spp. and Campylobacter spp. in the West Bank, Palestine. Appl. Environ. Microbiol..

[48] World Health Organization United Nations Environment Programme, World Organisation for Animal Health (2022 Oct 14).

[49] Roth F., Zinsstag J., Orkhon D., Chimed-Ochir G., Hutton G., Cosivi O. (2003). Human health benefits from livestock vaccination for brucellosis: case study. Bull. World Health Organ..

[50] Bucher A., Dimov A., Fink G., Chitnis N., Bonfoh B., Zinsstag J. (2023). Benefit-cost analysis of coordinated strategies for control of rabies in Africa. Nat. Commun..

