# Supplementary material for: Implementing one health in Palestine: Mapping ministerial mechanisms for pandemic preparedness, zoonotic disease control, and inter-sectoral collaboration
Source: One Health. 2025 Jun 5;20:101100. doi: 10.1016/j.onehlt.2025.101100 (PMC12179705; doi:10.1016/j.onehlt.2025.101100)
Supplement: Supplementary file 1 — Supplementary material1 [file mmc1.pdf]

Supplementary Material 1: Stakeholders Participating in the Project Phases

Institutions	Departments\Position	Number
**Experts that Participated in Determining Endemic ZDs in Palestine**		**25**
Ministry of Health	Environmental Health Department	3
	Public Health Lab	
	Preventive Medicine Department	
Ministry of Agriculture	Head of Veterinary Control and Quarantine Department	7
	Veterinarian	
	Head of Laboratory Department	
	Veterinary Services Department	
	Director of Epidemiology Department	
	Head of Pathology Department and Quality Officer	
	Director of veterinary service	
Military Medical Services	Preventive Medicine Department	1
Birzeit University	Clinical Laboratory Sciences	4
	Department of Biology and Biochemistry	
	Associate Professor	
	Director of testing laboratory center	
Al-Najah University	Department of Veterinary Medicine	5
Al-Quds University	Associate Professor of Hematology	
Other Experts	Clinical Microbiologist	
Hospital		
Hospital	Head of Infectious Diseases Department	
UNRWA	Field Diseases Control Officer	3
WHO-Jerusalem	IHR/CO OFFICER	
FAO	Project Manager	
Hebron Municipality	Head of Health Control Department	2
Al-Bireh Municipality	Central Abattoir Doctor	
**Workshop on 16\8\2023 - Prioritizing Zoonotic Diseases**		**36**

Ministry of Health	Environmental Health Department	12
	Preventive Medicine Department	
	Director of Paramedic	
	General Administration of Primary Health Care	
	Dermatologist	
	Central Public Health Laboratory	
	General Directorate of Public Health	
	Quality Department	
Ministry of Agriculture	General Directorate of Veterinary Services amd Animal Health	11
	Public Health Department	
	Epidemiology Department	
	Veterinary Laboratories Department	
	Veterinarians	
Environment Quality Authority	Environmental Health Department	3
Military Medical Services	Preventive Medicine Department	1
FAO	Head of Programs	4
	Program Manager	
WHO	IHR/CO Officer	
UNRWA	Field Diseases Control Officer	
Al-Bireh Municipality	Central Abattoir Doctor	1
Al-Najah University	Department of Veterinary Medicine	4
Birzeit University	Lab technician	
Hospital	Infectious Disease Specialist	
Other Experts	Clinical Microbiologist	
**Workshop on 17\4\2024 - Mapping on Networks**		**23**
Ministry of Health	Environmental Health Department	12
	Preventive Medicine Department	
	Preventive Medicine Nursing Department	
	General Administration of Primary Health Care	
	Central Public Health Laboratory	
	Director of Molecular Biology and Genetics Department	
	Microbial Bioanalysis Department	
	Head of Water Devision	

	Water testing Unit	
	Pest Control Department	
	Health Directorate of Jericho - Nurses	
Ministry of Agriculture	Veterinary Laboratories Department	7
	Veterinarians	
Military Medical Services	Preventive Medicine Department	1
UNRWA	Field Diseases Control Department	1
Al-Bireh Municipality	Central Abattoir Doctor	1
Birzeit University	Lab technician	1
