# Supplementary material for: Implementing one health in Palestine: Mapping ministerial mechanisms for pandemic preparedness, zoonotic disease control, and inter-sectoral collaboration
Source: One Health. 2025 Jun 5;20:101100. doi: 10.1016/j.onehlt.2025.101100 (PMC12179705; doi:10.1016/j.onehlt.2025.101100)
Supplement: Supplementary file 2 — Supplementary material2 [file mmc2.pdf]

Supplementary Material 2

Prioritizing Zoonotic Diseases Adapted Tool – Discussion Group Guide

Guideline /Question:

1. What is the Case -Fatality Rate (CFR) of the disease in humans? (Criterion weight **= 0.24**)

a. Disease present, high CFR (≥5%) (score = 1)

b. Disease present, low CFR (less than 5%). (score = 0.67)

c. Disease not known to present, high CFR (≥ 5%) (score = 0.33)

d. Disease not known to be present, low CFR (less than 5%) (score = 0)

2. What is the proportion of human disease attributable to animal exposure? (Criterion weight **= 0.22)**

a. Sustained animal-to-human transmission (no human-to-human) (score = 1)

b. Human-to-human transmission possible, but not sustained (score = 0.50)

c. Human-to-human sustained transmission (score = 0)

3. What is the financial burden of the disease on humans? (Economic impact) (Criterion weight: **0.12**)

a. Expensive medication with hospitalization (score = 1)

b. Expensive medication without hospitalization (score 0.67)

c. Moderate medication cost (score = 0.33)

d. Self-cured without medication (score = 0)

4. What is the financial burden of the disease on animals?) criterion weight = 0.1)

a. Disease present with loss of production (score=1)

b. Disease present, with unknown loss of production– (score 0.67)

c. Disease present with no loss of production (score = 0.33)

d. Disease not present with no loss of production (score = 0)

5. Which prevention and control measures are currently available in Palestine? (Weight = **0.15**)

a. Animal vaccine (score = 1)

b. Human intervention (vaccine or treatment) (score = 0.50)

c. Neither (score = 0)

6. Is there currently any inter-sectoral collaboration? (Criterion weight = 0.17) (Please ask the interviewee to specify with whom/ between what entities)

a. Yes, current strong collaboration (score = 1)

b. Yes, previous or weak collaboration (score = 0.5)

c. No (score = 0)

Weighted Score Calculation for Each Disease

Disease weights were calculated by multiplying response scores by criterion weights and summing the results for each disease, as expressed in the formula below. The average weight across groups was then determined. Stakeholder insights were incorporated to refine the prioritization of zoonotic diseases (ZDs).

$$W_i = \sum (S_{ij} \times C_j)$$

Where:
- W_i: Weighted score for disease i
- S_{ij}: Response score for disease i according to criterion j
- C_j: Weight assigned to criterion j
- n: Total number of criteria used in the prioritization

Average Weight Calculation Across Groups:

The average weight for each disease across all stakeholder groups is calculated as:

$$\bar{W}_i = (\Sigma\, W_{ik}) / m$$

Where:
- $\bar{W}_i$: Average weight for disease i

- W_{ik}: Weighted score for disease i in group k
- m: Total number of stakeholder groups
