# Supplementary material for: Implementing one health in Palestine: Mapping ministerial mechanisms for pandemic preparedness, zoonotic disease control, and inter-sectoral collaboration
Source: One Health. 2025 Jun 5;20:101100. doi: 10.1016/j.onehlt.2025.101100 (PMC12179705; doi:10.1016/j.onehlt.2025.101100)

Supplementary Material 3: List of zoonotic diseases and the percentage distribution of experts' responses on each disease

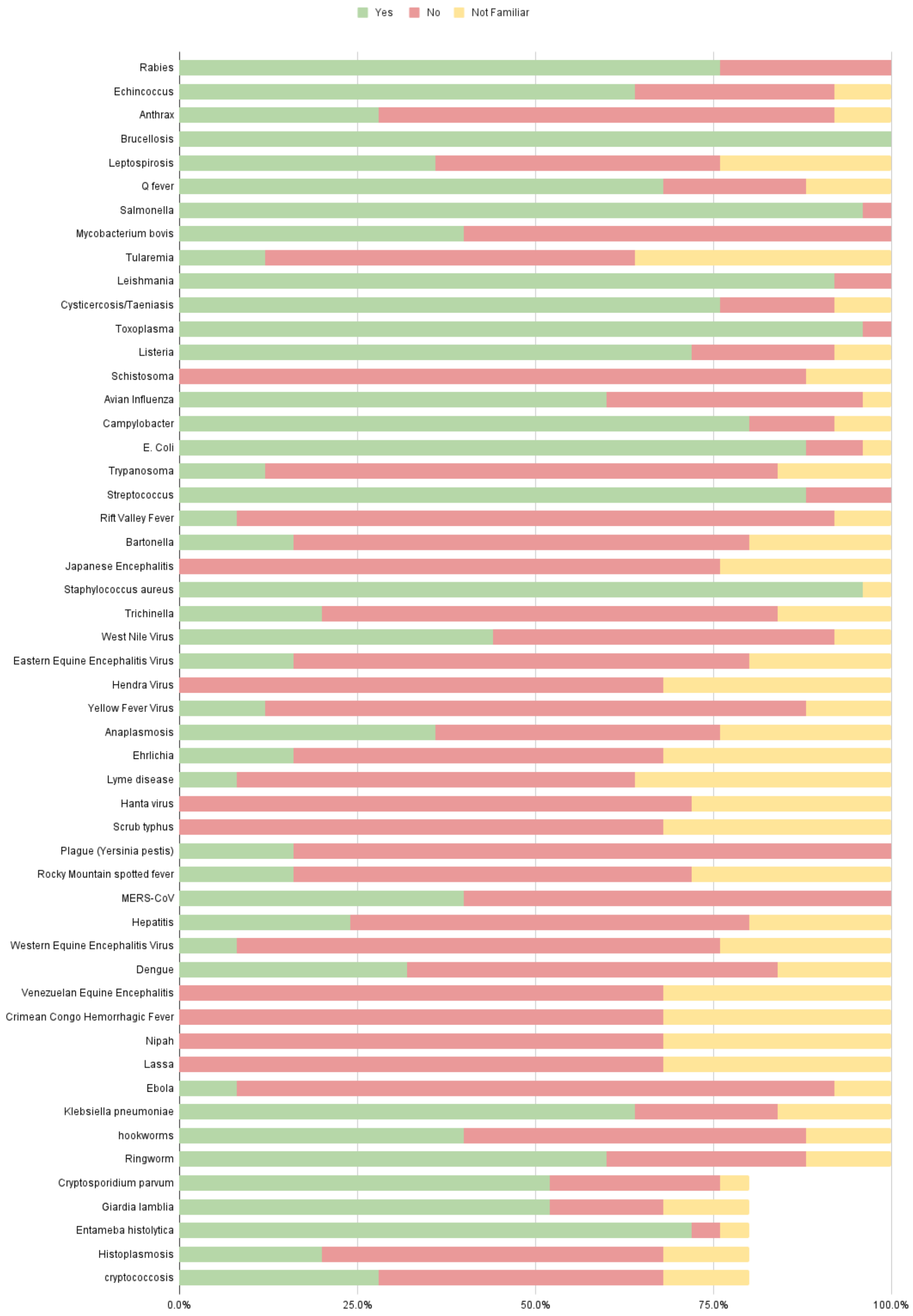

Supplement: Supplementary file 3 — Supplementary material3 [file mmc3.pdf]
