# Supplementary material for: Implementing one health in Palestine: Mapping ministerial mechanisms for pandemic preparedness, zoonotic disease control, and inter-sectoral collaboration
Source: One Health. 2025 Jun 5;20:101100. doi: 10.1016/j.onehlt.2025.101100 (PMC12179705; doi:10.1016/j.onehlt.2025.101100)
Supplement: Supplementary file 4 — Supplementary material4 [file mmc4.pdf]

Supplementary Material 4: Abbreviation’s list:

CDC: Centers for Disease Control and Prevention

CPHL: Central Public Health Laboratory

CVL: Central Veterinary Laboratory

FAO: Food and Agriculture Organization of the United Nations

iSRS: Integrated harmonized surveillance–response system

LMICs: Low and middle-income countries

MoA: Ministry of Agriculture

MoH: Ministry of Health

NGO: Non-governmental organizations

PHC: Primary Health Center

PMD: Preventive Medicine Department

RRT: Rapid Response Teams

UNRWA: United Nations Relief and Works Agency for Palestine Refugees in the Near East

WHO: World Health Organization

WOAH: World Organization for Animal Health

ZDs: Zoonotic Diseases
